# A SEM-NCA Approach towards Social Networks Marketing: Evaluating Consumers’ Sustainable Purchase Behavior with the Moderating Role of Eco-Friendly Attitude

**DOI:** 10.3390/ijerph182413276

**Published:** 2021-12-16

**Authors:** Pejman Ebrahimi, Datis Khajeheian, Maria Fekete-Farkas

**Affiliations:** 1 Doctoral School of Economic and Regional Sciences, Hungarian University of Agricultural and Life Sciences (MATE), 2100 Gödöllő, Hungary; Ebrahimi.Pejman@stud.uni-mate.hu; 2 Department of Business Management, University of Tehran, Tehran 1411713114, Iran; 3 Institute of Economic Sciences, Hungarian University of Agricultural and Life Sciences (MATE), 2100 Gödöllő, Hungary; farkasne.fekete.maria@uni-mate.hu

**Keywords:** consumers’ sustainable purchase behavior, social networks marketing, social media marketing activities, eco-friendly attitude, SEM-NCA

## Abstract

This paper aims to investigate how social network marketing affects consumers’ sustainable purchase behavior (CSPB) while considering the role of Eco-friendly attitude. The statistical population of the study included Iranian users of online social networks with at least one online purchasing experience. An online questionnaire was distributed on Instagram, Telegram, and WhatsApp platforms as the most popular networks in the country. By use of convenience sampling, commonly used in quantitative studies to overcome bias, 450 out of 475 returned questionnaires were acceptable, showing a response rate of 94.7%. The results indicated that an increase in Eco-friendly attitude positively increases the effect of word of mouth on consumers’ sustainable purchase behavior. Meanwhile, Necessary Condition Analysis (NCA) revealed that to reach a 50% level of consumers’ sustainable purchase behavior, six essential necessary conditions are required: an eco-friendly consumers’ attitude at no less than 50%, the trend at no less than 57.1%, word of mouth at no less than 45.5%, interaction at no less than 42.9%, customization at no less than 35.3% and entertainment at no less than 26.7%. Furthermore, the Importance-Performance Matrix Analysis (IPMA) was investigated as a strategic tool. The results of IPMA showed that “buy products that use biodegradable material in packaging”, “buy those products that are picked up and recycled”, and “buy biodegradable products even if they belong to a less well-known company” show desirable performance and high importance and there is a great opportunity for expansion in this area.

## 1. Introduction

Social media have become the venue for various aspects of everyday life and people spend a large amount of their time on these platforms [1]; therefore customers’ behaviors in these networks are of high importance for business owners as well as policymakers [2]. With further growth of global access to the internet, in addition to investment in mass media and television advertising, companies and brands use social networks as a new platform and business opportunity [3,4,5]. Research shows that the promotion of goods and services on social media is more effective in the long run [6,7,8]. Social networks marketing builds strong customer relationships because it reaches participatory audiences and engages customers [9,10,11]. Social media users share and consume a product or brand-related content to educate each other about the company’s sustainable practices and supply chain [12,13]. Sharing these personal experiences and opinions on social media increases word of mouth (WOM) [13,14]. With the increasing use of social media, more data are generated and new opportunities are created to analyze several aspects and communication patterns. For example, social media data can be analyzed to obtain information about preferences, trends, influential actors, and other types of useful data [3,15,16]. Over the years, with the significant increase in the number of internet and social media users, the patterns of consumer behavior have changed around the world, which has led to a changing trend in consumer readiness to seek information through new mass communication channels [17]. According to the International Telecommunication Union (ITU), about 4.66 billion people, over half of the world’s population, have access to the internet and about 4.2 billion are active social media users [2]. Due to the ever-increasing exponential use of the internet and social media, they have become the primary source of business-related information about products and brands [14]. Therefore, interaction or communication with other users and content created by an organization, company, or person highlights the importance of using social media [18,19]. To build sustainable performance and strong relationships, the production of marketing content in a social media strategy framework is an important challenge for companies because they must align marketing content with the customer’s personal or community preferences [20].

On the other hand, many countries, particularly developing ones, face high levels of air pollution, low water quality, high levels of traffic noise pollution, high levels of non-disposable waste, and rapid depletion of energy resources. Abnormal consumption and dangerous activities are the leading causes of environmental problems [21]. Iran as a developing country suffers from such challenges too. A study of global statistics and the world population in 2021 shows that, with an annual production of about 4 million tons, Iran is ranked 17th in the production of plastic waste. Due to an increase in environmental concerns and the negative effects of abuse on human physical and mental health, encouraging people to consume eco-friendly products is a top priority in many countries’ industrial policy [22], and it is not surprising that social networks are one of the most important tools to encourage consumption of such products. Therefore, investigating how social networks marketing can affect Consumers’ Sustainable Purchase Behavior is an important matter. A review of the literature revealed that, in the context of Iran, there is little research on the subject of consumers’ sustainable purchase behavior. Considering investment within the country in the production of eco-friendly products, a study of how social network marketing as an eeffective contemporary tool can promote eco-friendly attitudes and therefore enhance consumers’ sustainable purchase behavior is highly needed by the country’s policymakers to encourage people towards more sustainable purchase behavior.

To date, there has been little comprehensive research on the effect of social network marketing on consumers’ sustainable purchase behavior. To fill this gap, the present study has adopted an eco-friendly attitude as the main driver of sustainable purchase behavior considering it as the moderator variable. The research question (RQ) is ‘How can social networks marketing affect consumers’ sustainable purchase behavior regarding the eco-friendly attitude of consumers?’. To answer this question, the NCA method is used as an innovative method to know how different dimensions of social media affect consumer behavior at different levels. The overall structure of this study takes the form of five sections. Throughout the paper, after an introduction, a literature review is presented, where the research variables are conceptualized and the links between them are established to develop the hypotheses. Then, the methodology is introduced and the results of data analysis are discussed. To conclude, theoretical and practical contributions are issued and finally limitations and suggestions for future research are presented.

## 2. Literature Review and Hypotheses Development

Social media have brought transparency and revolutionized the way consumers interact with each other [14,17]. The use of social media as a marketing channel by a company or individual should provide marketing services, techniques, strategies, and plans that demonstrate social participation and meet the characteristics of the community [23]. In addition to relying on commercially-oriented aspects, social networks marketing content should also be socially oriented or involve active interaction between users [24] and create deep communication and good relationships between them [25]. This increase in transparency gives consumers more access to information related to the use of the product and increases awareness of the conditions under which industrial corporations produce them. In addition to their growing sales of sustainable products [26], this puts pressure on organizations to address environmental issues and the social sustainability resulting from their performance and adopt sustainability practices throughout their supply chain network [27]. The internet and social media change consumer behavior and increase environmental awareness [28], consequently increasing sales of green products [29] and promoting green behavior and loyalty to products and brands [30,31]. Thus, environmental issues become more frequently considered by consumers and they are more aware of the consequences of the effects of consumption on the environment [2,32]. These activities incline companies to develop and produce environmentally friendly products that are less harmful to the environment [33].

### 2.1. Social Networks Marketing

The emergence of social media such as Facebook and Twitter [34] has provided an opportunity to create, share and receive information at a low cost. A study shows that adults spend about 6 h daily on social media and often use multiple platforms simultaneously [35]. Social media create communities that interact with and mutually influence each other [36]. Organizations also take full advantage of the opportunities provided by social networks [37]. Social media are a marketing tool with a different appeal than traditional marketing platforms such as print advertising, billboards, etc. [38]. According to Carr and Hayes [39], social media are “Internet-based channels that allow users to easily and selectively interact with each other and gain value from user-generated content” [40]. Based on Mangold and Fowled [41], social media are divided into 15 types, including social networking sites, creativity sharing sites, user-sponsored blogs, etc. Kaplan [42] and Haenlein argues that social media are characterized by the features of Web.2.0 technology, including user-generated content, information exchange, and social networking, thus creating a high degree of social interaction [43].

One of the foundations of market success is effective marketing communications. Exceeding the processing capacity of the number of communication messages by target market, rationalization, and effective targeting are the main factors in the final survival of the transmitted message [44]. The integrated activities of organizations that are related to the social media marketing strategy lead to the desired achievement of communication (networks) and interactions (influences) to achieve the desired effective marketing [45]. Companies advertise products through social media, where brands control and promote their e-reputation by partnering with the public. Brands contact brand prescribers, influencers, or brand ambassadors to support a product or service. Therefore, using this business strategy, maximum visibility is created with minimum investment [46].

### 2.2. Consumers’ Sustainable Purchase Behavior

Consumer purchase behavior determines the analysis of customer behavior, assuming that the customers play the role of distinct users, the payer, and the buyer [47]. There are four types of buying behaviors: complex purchase behaviors, buying behaviors that seek diversity, buying behaviors that aim to reduce post-purchase stress, and normal buying behaviors [48].

- The prevailing approach, which explains the principles of consumer behavior, describes the consumer buying process as learning, information processing, and decision-making activity that is divided into five stages: Problem identification; Information search; Alternatives evaluation; Purchasing decision; Post-purchase behavior [49,50,51,52,53,54].

Due to changes in culture, economics, and technology, consumer behavior is diverse and comprehensive [47]. Purchasers’ behavior, in addition to their desire to meet individual needs, also includes concern for the interests of the entire society and for environmental issues [55].

The impact of people’s consumption patterns on society and the planet is generally ignored, while irresponsible consumption patterns lead to “natural, social and economic destruction” [56]. In efforts to increase consumption and modernization, damage to the environment and society is often overlooked [57]. Indeed, sustainable choices involving long-term benefits for other people and the natural world should take precedence over the typical consumer decision that classically focuses on maximizing immediate benefits for oneself. Despite the usefulness of comprehensive marketing strategies in this area, marketers need to develop a unique set of tools to promote sustainability [58].

Marketers try to facilitate sustainable consumer behavior. According to Ripple et al. [59], “Marketers need to know that the consumerist mindset that encourages conventional marketing is the main driver of negative environmental impacts” [60,61]. According to Anderson, businesses that can adapt to the demands of our changing world, including the immediate need for sustainability, will have strategic benefits and will grow further in the long term [62].

Purchasing sustainable products that have social, economical, and environmentally friendly characteristics constitutes sustainable purchasing [63]. Green products are sustainable and environmentally friendly and limit their environmental effects throughout their life cycle. Reducing waste and maximizing the efficiency of valuable resources are the two essential dreams of sustainable products. The use of sustainable products leads to reducing or preventing environmental degradation by consumers [64].

### 2.3. Framework of Social Networks Marketing and Consumers’ Sustainable Purchase Behavior

The framework of this research is taken from Social Media Marketing Activities (SMMA), including entertainment, interaction, trendiness, customization, and word of mouth [38].

Agichetein et al. [65] argue that entertainment is the pleasure originating from the social media experience. The use of social media, especially when gamification techniques come into use, provides fun and play for the users and this provides returning users with loyalty and purchasing intention [34,35,66,67]. Kim and Ko [38] believe that entertainment positively affects consumer attitudes and leads to the development of more interaction between brands and consumers.

**Hypothesis** **1** **(H1).**  *Entertainment stemming from social network marketing positively influences consumers’ sustainable purchase behavior*.

Customization implies that a product or service satisfies the customers’ preferences, needs, and demands [68]. In social media marketing, customization reflects how much messages, information, and advertising material agree with what customers are seeking [69,70]. Using customization, brands reflect the uniqueness of their products and services for their customers and promote their loyalty to the brand [71]. Customization is effective for companies because it allows customers to design and customize products and increases customer engagement. Similar services are provided to customers through companies such as Gucci and Burberry [72].

**Hypothesis** **2** **(H2).**  *Customization stemming from social network marketing positively influences consumers’ sustainable purchase behavior*.

Brahmmati and Ahmad [73] point out that the ultimate consumer purchase behavior of people who frequently use social networking sites for seeking information is influenced by social media marketing. Social networks provide space for users and customers to share their thoughts and exchange their purchasing experiences among brand-elated services and goods. Interaction among users on social platforms offers them insights [74]. The interaction within social networks has fundamentally changed the dynamic of communications between brands and customers via user-generated content [75,76]. Zhao et al. [77] reported significant social effects of Chinese consumer interaction with social media and peers, directly related to the increase in sustainable clothing purchase targets. According to Xhema [78], there is a two-way relationship between social media and customer behavior, where customers are less tolerant of poor service or overpriced prices.

**Hypothesis** **3** **(H3).**  *Interaction stemming from social network marketing positively influences consumers’ sustainable purchase behavior*.

Nasir et al. [79] showed that women consider traditional word-of-mouth advertising to be more credible than social media advertising in clothing purchasing decisions in Pakistan. The importance of social media in consumer attitudes, mental norms, altruistic and selfish motives was observed in the studies of Pop et al. [80]. According to their results, external factors such as sharing information on social media, a modern version of word of mouth, play an essential role in motivating the consumer to buy green cosmetics. E-WOM is “the credibility of product position information in a brand, which depends on the willingness and stability of companies to fulfill their promises” [81]. Ghafourian Shagardi [82] acknowledged the effectiveness of the dimensions of social networks, including electronic word of mouth marketing (eWoM), online advertising, and online communities in promoting brand loyalty and purchase intention.

**Hypothesis** **4** **(H4).**  *Word of mouth stemming from social network marketing positively influences consumers’ sustainable purchase behavior*.

Trendiness is a social media engagement tool that provides customers with the latest information on the latest trends [83]. Many users acquire their knowledge about brand services and products from what their friends and connections share on social media. Their attitudes about what products are recent and what services are worth trying and purchasing are obtained from social media [84]. According to the Madni Report [85], social networks play an essential role in consumer behavior when accessing information. For this reason, trendiness impacts the attitudes of customers regarding products and services.

**Hypothesis** **5** **(H5).**  *Trends stemming from social network marketing positively influences consumers’ sustainable purchase behavior*.

### 2.4. The Moderating Role of Eco-Friendly Consumers’ Attitude

Environmental pollution has increased consumers’ concerns about ethics, which has led to an increasing market ratio for sustainable products [86]. It is a widely held view among researchers and environmental activists thatconsumers can significantly improve the quality of the environment by purchasing environmentally friendly products, or green products, products with recyclable packaging, or proper disposal of non-biodegradable waste [87]. Therefore, all types of organizations must follow sustainable practices and use environmentally friendly packaging or green packaging in their work. This leads to the creation of healthy and safe communities and is also appropriate in terms of performance, cost, etc. [88]. It can also help consumers participate in environmental care activities, including (I) showing no inhibition in using recycled products, helping in the process of recycling the packaging or the used products, saving energy through using fewer energy-consuming appliances, (II) acceptance of lower technical performance of the product purchased due to its better eco-performance, (III) purchase of CFC free, biodegradable and organically produced products and (IV) willingness to 0pay a premium on eco-friendly products, and a 00tendency to prefer eco-friendly service provider in the areas of hospitality and tourism [89].

Product preparation using eco-friendly ingredients and eco-friendly packaging are two standard and different methods for producing eco-friendly products. Most manufacturers offer eco-friendly products using only one of the above methods, even if both methods can be used. This can be attributed to the cost of production and the possibility of using eco-friendly components, which leads to changes in the product’s primary characteristics, such as taste or other properties [90]. The eco-friendliness of materials is an internal feature of the product because environmentally friendly materials are directly related to the product itself. Organic products have the most environmentally friendly compounds [91]. The eco-friendliness of packaging is an external feature of the product because the packaging itself is an external element of the product. One of the essential elements for sale is packaging [92].

Schultz and Zelseny [93] argue that “attitudes of environmental concern are rooted in a person’s concept of self and the degree to which an individual perceives him or herself to be an integral part of the natural environment” [94]. An attitude is a belief about a particular behavior that can be positive or negative [95]. Ajzen [96] believes that if a person has a positive attitude towards that specific behavior, he is more likely to behave in a certain way. The subjective norm includes the individual’s interaction with a particular behavior due to perceived behavioral expectations and social pressure [80].

The level of knowledge, attitudes, values, and practices of consumers largely determines the environment’s quality [97]. Consumer environmental attitudes and behaviors are recorded in combination but are the vital concept needed to address the profile of environmentally conscious consumers. Attitudes are the most consistent explanatory factor in predicting consumers’ willingness to pay for green products [98]. Therefore, environmental attitudes include beliefs, desires, feelings, and behaviors related to the environment. Environmentally concerned consumers buy products and services with more positive (or less negative) impacts on the environment. These people consciously strive to save energy, seek to limit products made from scarce resources, and do not buy products with extra and inappropriate packaging [99]. Environmentally conscious consumers prefer to purchase products that are eco-friendly and biodegradable [100].

Various studies have investigated the impact of demographic and psychological dimensions on consumers’ environmental attitudes. Several lines of evidence suggest that there is a positive relationship between psychological variables and the environmental attitudes of consumers. A positive association has also been reported between favorable environmental attitudes and positive purchase decisions [101]. Consumers prefer to use ecologically packaged products, while non-recyclable packaging negatively affects their attitude towards using such products [88]. Having a positive attitude towards the environment causes consumers to buy more ecological clothes [102]. Considering the previous literature and research related to eco-friendly attitude, these moderating hypotheses have been expressed:

**Hypothesis** **6** **(H6a).**  *Eco-friendly consumers’ attitude moderates the effect of entertainment stemming from social network marketing on consumers’ sustainable purchase behavior*.

**Hypothesis** **6** **(H6b).**  *Eco-friendly consumers’ attitude moderates the effect of customization stemming from social network marketing on consumers’ sustainable purchase behavior*.

**Hypothesis** **6** **(H6c).**  *Eco-friendly consumers’ attitude moderates the effect of interaction stemming from social network marketing on consumers’ sustainable purchase bfehavior*.

**Hypothesis** **6** **(H6d).**  *Eco-friendly consumers’ attitude moderates the effect of word of mouth stemming from social network marketing on consumers’ sustainable purchase behavior*.

**Hypothesis** **6** **(H6e).**  *Eco-friendly consumers’ attitude moderates the effect of trends stemming from social network marketing on consumers’ sustainable purchase behavior*.

**Figure 1:**
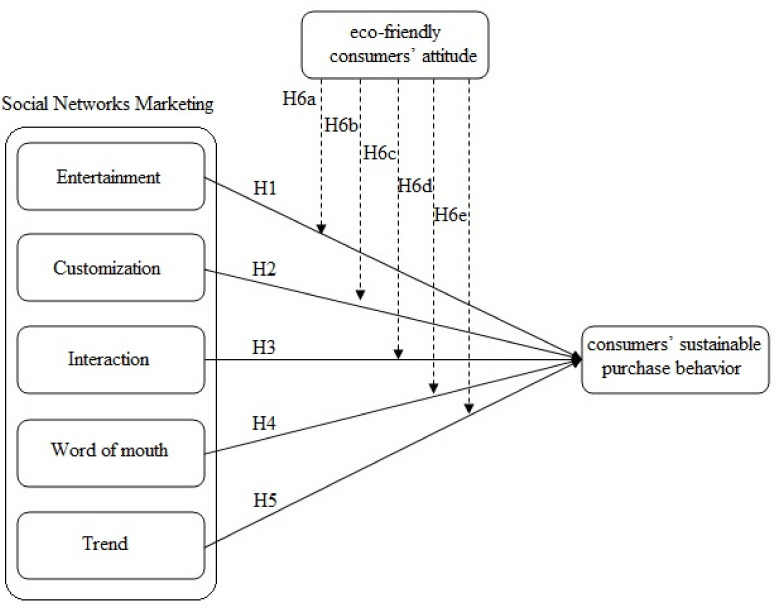
Research Model (Source: Authors).

Research model is presented in Figure 1.

## 3. Measures and Data Collection

**Figure 2:**
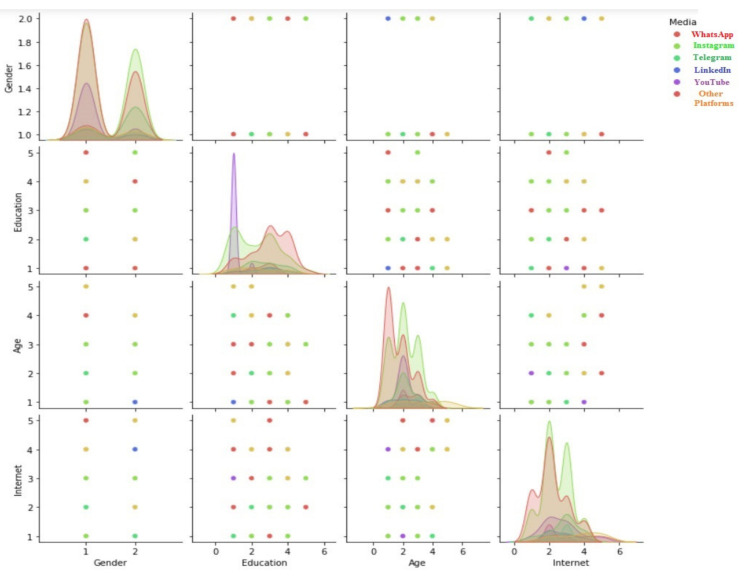
Pair plot of the respondents’ demographic data (Source: authors’ calculations based on Python programming/Seaborn package).

The first part of the questionnaire aims to gather information about the demographic data of the participants. In the research sample, 60% and 40% of the respondents were males and females, respectively. The majority of the respondents (44.5%) were 25 to 34 years old. Moreover, 40.2% of the respondents had a Bachelor’s degree, and 30.9% had a diploma or below diploma. Respondents were instructed to pay attention to the actual conditions while answering the questions with transparency and loyalty. A pair-plot of the respondents’ demographic data is depicted in Figure 2. According to this figure, based on time spent on social networks and different age groups, Instagram and WhatsApp are the most popular media. On the other hand, considering Education, YouTube is the most popular social network.

The second part of the questionnaire is designed to obtain data related to variables. The survey questionnaire items are adopted from previously tested scales. Social network marketing dimensions were measured using 18 items modified from a study by Kim and Ko [103]. We measured eco-friendly consumers’ attitudes by three items [104]. Finally, CSPB is measured by three items [88,105].

The indicators in the second part were measured using a 5-point Likert scale (1-strongly disagree, 5-strongly agree). The statistical population of the study involved Iranian users on online social networks who have made at least one online purchase Instagram, Telegram, and WhatsApp platforms were used to distribute questionnaires and online surveys. The reason for selecting these platforms is, as expressed before, their popularity in the country as the major social networks [106]. Online questionnaire links were shared among users on these online platforms. The results of another survey conducted by the Iranian Student Polling Agency (ISPA) show that 73.6% of Iranians over the age of 18 use social networks. This survey also shows that WhatsApp is the most popular social network in Iran among people over 18 years, with 64.1% users. After WhatsApp, Instagram ranks second with 45.3%, and Telegram ranks third with 36.3% of users [106].

Users were asked to answer the questions honestly. Since there were restrictions due to Covid 19 in Iran at the time of data collection, sending an online questionnaire link was the best way to collect data. The questionnaire link was shared for more than 90 days on the above-mentioned online platforms. Out of 475 distributed questionnaires, a total of 450 respondents successfully and correctly filled out the entire study with a response rate of 94.7%. We omitted questionnaires that were carelessly filled out (25 questionnaires).

This research uses the convenience sampling approach in gathering the data. This approach is commonly used in quantitative studies to overcome bias [107]. We have also employed the common method bias (CMB) test [108]. Harman’s single-factor has been carried out with seven variables to ensure that the collected data do not have CMB. The seven factors were then loaded into a single factor. The analysis shows that the largest variance explained by the newly created factor is 45.20%, which is below the threshold value of 50% [108]. Hence, there were no concerns regarding the CMB in the collected data. Furthermore, a pilot study was done to ensure the content validity and reliability of the sample size.

## 4. Results

**Figure 3:**
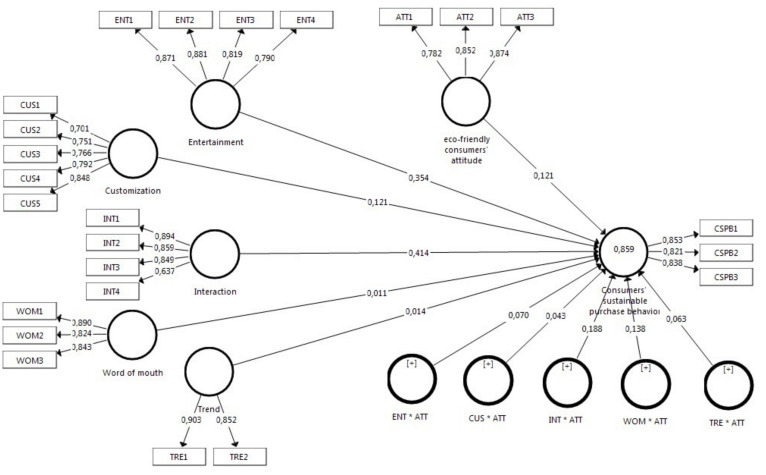
Path coefficients model (CUS: Customization, INT: Interaction, WOM: Word of Mouth, ENT: Entertainment, TRE: Trend, ATT: Eco-friendly Attitude, CSPB: Customers’ sustainable Purchase Behavior).

This study employs partial least squares structural equation modeling and the SmartPLS 3 software [109] to estimate and evaluate the research model (Figure 3). The analysis follows the guidelines, procedures, and critical values as presented by [110].

PLS-SEM presents satisfactory features upon dealing with complex models, non-normal data, and small samples [111]. Table 1 depicts via means and standard deviations that the variances in variables were significant. Accordingly, participants in this sample were substantially different in terms of the extent to which they perceived the significance of CSPB. Consequently, the sample was well usable for testing our hypotheses.

### 4.1. Evaluation of Measurement Models

Convergent validity was assessed by average variance extracted (AVE) scores and outer loadings. In SEM-PLS of SmartPLS software, Cronbach’s alpha, CR, and rho_A values higher than 0.7 are considered acceptable for internal consistency and reliability [113]. Furthermore, the value of average variance extracted at 0.5 shows acceptable and robust convergent validity [114,115], as this means that more than 50% variation in a specific construct is enlightened by the stipulated indicators [116] Meanwhile, all outer loadings are above the critical threshold of 0.7, which shows robust convergent validity (Table 1).

Table 2 depicts the result of the indicator multicollinearity with VIF. VIF values less than 3 are considered ideal values [117,118]. Discriminant validity was evaluated using the Fornell-Larcker criterion. The Fornell-Larcker criterion entails that the square root of the AVE for every construct should be higher than the inter-construct links [119]. Therefore, we conclude that discriminant validity has been established (Table 2).

### 4.2. Structural Model Assessment

The assessment of the structural model includes collinearity among constructs (evaluate multicollinearity between the independent variable constructs of the structural model), significance and relevance of the path coefficients, and the predictive relevance (e.g., R^2^, PLSpredict). We used common method variance (CMV). The highest VIF of the structural model has a value of 3.186. The method suggested by Kock [120] has been used [103]. If the occurrence of VIF is greater than 3.3, it is an indication that the model is contaminated with CMV and collinearity. However, in this study, all factor level VIFs resulting from the full test are lower than the recommended threshold 3.3, considering that the model is free from CMV. Therefore, CMV is not an issue in this study.

Significance testing uses bootstrapping with 5000 subsamples. Entertainment (β = 0.354, CI = (0.286; 0.451)), Customization (β = 0.121, CI = (0.463; 0.612)) and Interaction (β = 0.414, CI = (0.782; 0.869)) have positively significant effects on CSPB. Thus, H1 to H3 are supported (see Table 3). Word of mouth (β = 0.011, (CI = 0.767; 0.885)) and trend (β = 0.014, (CI = 0.644; 0.802)) with confidence level of 95%, have no significant effects on CSPB. Hence, H4 and H5 are not supported.

**Figure 4:**
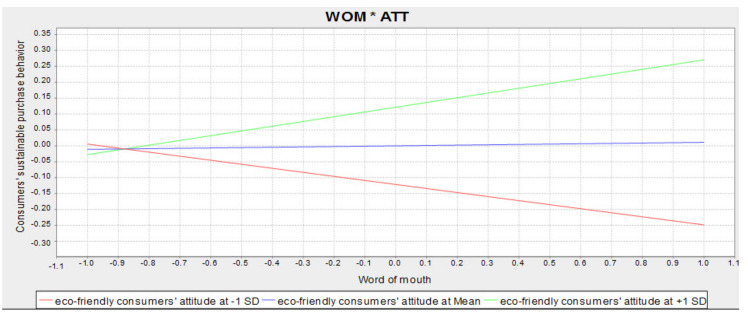
Moderating effect (Word of Mouth*Attitude-Consumer Sustainable Purchase Behavior).

The product indicator approach in SmartPLS3 was used to assess moderating effects. H6c (INT*ATT-CSPB) is supported (β = 0.188, (CI = 0.767; 0.885)). Likewise, H6d (WOM*ATT-CSPB) is also supported (β = 0.138, (CI = 0.767; 0.885)). Figure 4 illustrates that an increase in ATT positively increases the effect of word of mouth on CSPB. Other moderating effects (H6a-H6b-H6e) are not supported (see Table 3).

The model explained 79.7% of the variance in CSPB. Furthermore, the out-of-sample predictive power was determined using the PLSpredict procedure [112,121,122]. Q^2^ predict value of CSPB was above zero and acceptable. In fact, the linear model (LM) had a better root mean square error (RMSE) for all target construct indicators in comparison with the PLS-SEM benchmark (Table 4). Thereby, the model had high predictive power.

Meanwhile, model fit was assessed by evaluating the SRMR [123]. As the SRMR value for this research model was 0.079, lower than the threshold value of 0.08 [10], it can be concluded that the model is a reasonable model fit.

### 4.3. Necessary Condition Analysis (NCA)

Dul developed an NCA method in 2016 for the first time. Instead of analyzing the average relationships between dependent and independent variables, NCA shows areas in scatter plots of dependent and independent variables that could specify the presence of a necessary condition [124,125,126].

**Figure 5:**
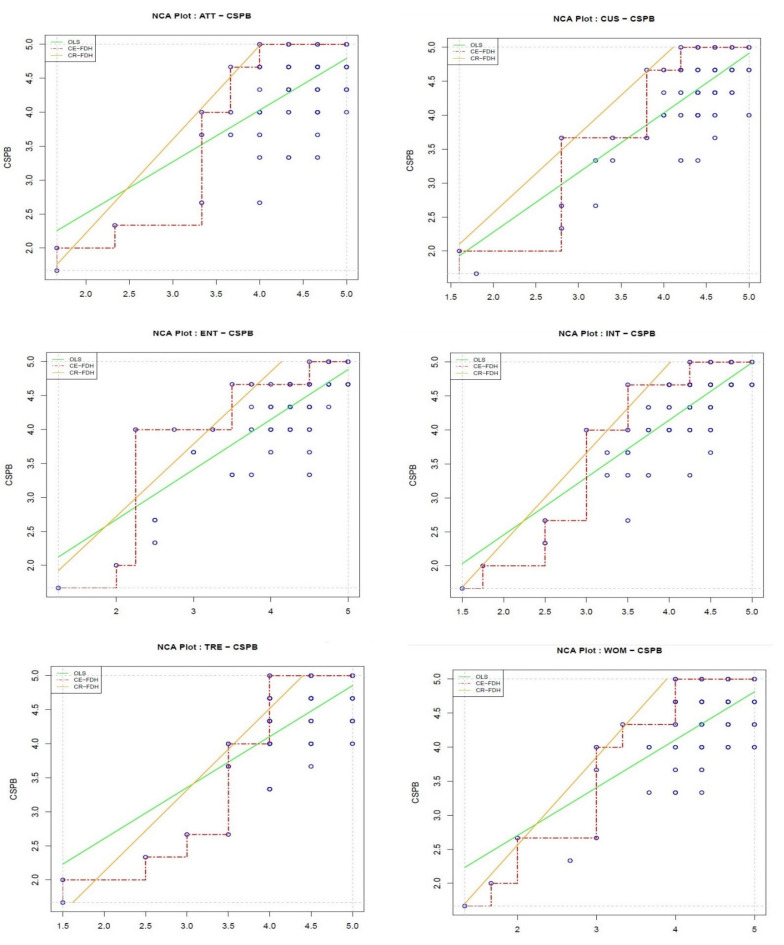
Scatter plots of Necessary Condition Analysis (NCA).

This study tried to show if entertainment, customization, interaction, word of mouth, trend, and eco-friendly consumers’ attitudes were necessary conditions for CSPB or not. Figure 5 displays the scatter plots for all relevant relations. Table 5 shows the effect sizes. As the accuracy of the ceiling envelopment-free disposal hull (CE-FDH) ceiling line is per definition 100%, a separate column was not added for the ceiling line accuracy.

The NCA’s results (see Table 5) specify that independent variables are meaningful (d ≥ 0.1) and significant (*p* < 0.05) necessary conditions for CSPB. It is possible to evaluate each necessary condition in detail with the bottleneck tables. For instance, Table 5 shows that to reach a 50% level of CSPB, six essential conditions are required: eco-friendly consumers’ attitude at no less than 50%, the trend at no less than 57.1%, word of mouth at no less than 45.5%, interaction at no less than 42.9%, customization at no less than 35.3% and entertainment at no less than 26.7%.

## 5. Discussion

The present study examined the moderating role of eco-friendly consumer attitudes in the relationship between social media marketing features and CSPB. The findings of this study can provide helpful insights for marketing activists, particularly social media marketers. The results show that if marketing managers pay attention to eco-friendly attitudes of consumers in content creation for social media, it will help them to achieve marketing goals.

According to the first hypothesis test, entertainment has a positive impact on CSPB. This result is in line with the result of research conducted by Hyun et al. [127] but inconsistent with the result of research conducted by Omidi et al. [128]. In fact, entertainment in the purchase of environmentally friendly products creates the intention to search and followingly the intention to purchase. Therefore, it can be argued that to introduce eco-friendly goods and services, entertainment can be considered a reference for designing social media pages and advertising on them. In fact, by creating an entertaining environment on social networks, growth hackers can provide exciting and enjoyable conditions to increase buyers’ enjoyment and encourage them to search and purchase eco-friendly products. Thus, due to confirmation of the first hypothesis, the marketing manager should produce advertising content or messages with an exciting story or exciting music. This content design should be such that the customer feels excited, happy, or entertained after watching it. The social media marketing environment should be able to increase consumers’ pleasure needs by providing enjoyable entertainment. The value of entertainment lies in its ability to improve users’ needs for emotional discharge by providing the opportunity to experience and to exchange information and consumers’ photos and videos with their social communications. To attract consumers to eco-friendly products through advertising, manufacturers should design advertising that motivates customers to use the products. In other words, the commercial advertisement should encourage potential consumers to purchase eco-friendly products via the publishing of information. Attracting people and showing the quality of products by creating positive feelings and a pleasant experience is one of the main functions of commercial advertising.

Confirmation of the second hypothesis of this study indicates that customization on social media has a positive impact on CSPB. This result is not in line with previous research [103]. The most important dimension in evaluating social media actions is customization. Brand owners need to create appropriate value to meet a customer’s need; otherwise, they cannot attract customers and encourage them to use their product or service. In fact, the proposed value can be the reason for preferring a product to other similar products on the market, which affects CSPB and leads to purchasing. Companies can offer value resulting from customization either quantitatively (easy accessibility, speed of service) or qualitatively (product customization). For example, a portal that provides an opportunity to make electronic foreign currency payments and issue foreign currency credit cards for tens of thousands of people is a company that could gain a good position among customers through the customization of valuable services. By confirming this hypothesis, it is also suggested that the layout of content and posts on social media should be made perceivable, transparent, and orderly by marketing managers so that customers can easily select the product information they want. They can use hashtags to help the customers to find the product information easily.

The third hypothesis of the study indicated that interaction on social media has a positive impact on CSPB. It can be stated that social buyers expand their social relationships by searching on social networks. They develop friendly relationships by talking and exchanging ideas and communicating, and exchanging information for their purchases by using this tool. Thus, brand managers should not use social media only as a marketing tool but should consider the design and planning of social media marketing programs as essential and use social media marketing as a source for interaction with consumers to gain in-depth information about their preferences, intentions, and behavioral patterns. Increasing relationships among members of social media will strengthen social interaction, meaning that members share more information through interaction with each other. Social interaction among members is the easiest way to access a significant portion of the information on or features of products because most consumers prefer to seek information about the product from other consumers to reduce the risk of uncertainty before purchasing specific products or services and thus improve CSPB.

Confirmation of the third research hypothesis of the study indicates that interaction has a positive effect on CSPB, and the transmission of opinions through social networks, especially about sustainable products and services, builds trust and strengthens this feeling in customers. This result is in line with the result of previous research [77,78,129,130,131]. It can also be stated that the interaction of social media users with other users enables them to form their perception of purchasing sustainable products. According to this hypothesis, social media marketers are recommended to consider that online consumers that use social media are more likely to purchase from them. They should choose platforms for their advertising that allow presentation and exchange, and general two-way interaction for customers. For example, there are features for interaction in the Instagram story, such as Q&A and multiple-choice questions, that companies and brands can use to communicate with their consumers. The results obtained regarding these hypotheses are consistent with the results of Wibowo et al. [20], Pop et al. [80], Xhema [78], Abbas et al. [132], Cetina et al. [133], Khodadad Hosseini et al. [134], Madani [135], Brahmati and Ahmad [73] Ioanăs and Stoica [136], and Fagerstrom and Gheina [137], because they also believed that social media marketing and its elements have an impact on CPB.

The results of H5 are in line with the result of research conducted by Kim and Ko [83], but the result of H4 is inconsistent with the result of research conducted by previous researchers [79,130].

The results of this study regarding the H6c and H6d hypotheses showed a significant association between interaction and word of mouth with the CSPB, emphasizing the potential moderation impact of eco-friendly consumers’ attitudes [88,96,100,102]. This type of content increases consumers’ attitudes towards the environment. It encourages them to participate in the promotion of this type of product, and gradually this attitude spreads among other users and consumers. An important point in the H6d hypothesis is that, regardless of the moderation role of eco-friendly consumers’ attitude, word of mouth has no impact on CSPB, showing this lack of relationship with the rejection of the fourth hypothesis. Thus, it can be stated that eco-friendly consumers’ attitude moderates the effect of word of mouth on CSPB. One of the reasons that have caused word of mouth to affect the CSPB through a friendly consumer attitude is that they have tended to consume products that cause less environmental pollution than other similar products in recent years. Marketing managers can emphasize the eco-friendly feature of the product or service. They can also expand the consumption attitude of eco-friendly products, inform users about the concerns and effects of environmental pollution in producing their content, and warn them about the influence of environmental pollution on human health.

## 6. Conclusions

Using social media to attract new buyers and retain existing consumers is crucial in marketing activities due to increased competition among brands. By offering many alternatives to customers and intensifying competition between brands, they should take positive steps to increase sales and profits by providing new products to customers on social media. Given that the core of marketing is consumer attitudes, the success or failure of existing and new market products and services in the CPB is determined by their sustainable performance. The purpose of this study is to investigate the impact of social media marketing on CSPB, considering the moderating role of eco-friendly consumer attitudes. The results of previous studies in this field were identified. Five characteristics of social media marketing (entertainment, customization, interaction, word of mouth, and trends) as factors affecting CSPB, so the first to fifth hypotheses of this study were developed.

In the present study, the IPMA matrix was investigated as a strategic tool. The results showed that by targeting the CSPB variable, in terms of performance, all dimensions of social networks marketing are in a competitive and desirable position in Iran to affect CSPB. Interestingly, the eco-friendly consumers ’attitude variable has the most desirable performance in the statistical population of the present study. However, it needs to pay more management attention in terms of importance. In fact, the items of “buy products that use biodegradable material in packaging”, “buy those products that are picked up and recycled”, and “buy biodegradable products even if they belong to a less well-known company” have desirable performance.

The interaction variable has the highest importance compared to other variables, while it has lower performance compared to other constructs. In fact, “exchange opinions”, “conveying opinions”, “two-way interaction”, and “sharing data” have great importance from the perspective of respondents. According to the results, entertainment plays a vital role in influencing CSPB, and more attention needs to be paid to this variable in the study area.

### 6.1. Managerial Implications

Valuable management information is obtained from the results of the NCA approach. Given the need for business owners to achieve different levels of CSPB from other structures, this is an essential strategic finding for the appropriate allocation of resources. This is really important and practical information for making reliable and valid decisions. NCA analysis shows, to reach a different level of CSPB, how many essential conditions are required. Managers can focus on different levels of NCA analysis to manage resource allocation and finally can increase efficiency and productivity, especially in online businesses.

From a strategic and management perspective, the ENT variable requires the most investment from online businesses. Although all research variables play a significant role in explaining CSPBs, allocating resources is an essential point that the NCA approach has responded to and can provide a better management perspective along with the IPMA matrix.

Based on IPMA matrix results, regarding SNM dimensions, “Two-way interaction through social networks” has the highest importance score; if businesses augment their interaction by one unit point, the overall CSPB will increase even more than before. Furthermore, our results display that the lowest performance related to this scale can offer a great opportunity for expansion in this area. “Customized services” shows a second scale need for more managerial attention and can increase CSPB regarding IPMA matrix results.

In terms of variable level, “eco-friendly consumer attitude” has the highest importance score and needs special attention. Consumers are interested in “eco-friendly packaging” based on collected data. This shows a great opportunity for businesses to invest in eco-friendly products.

### 6.2. Limitations and Suggestions

The present study suffers several limitations. In this study, data were collected through a cross-sectional approach during a specific period of the Covid 19 epidemic. It is suggested for future studies that a longitudinal approach be used. In addition, we should tread with caution in generalizing the research results, since the respondents of this research answered the questionnaire questions based on the experience of using online products and services in Iran. In other countries or cultures, different results or experiences might be observed. Future studies can also examine the moderating role of demographic variables in SmartPLS 3 software. It is also suggested to use the FIMIX approach for future studies to uncover unobserved heterogeneity in the internal (structural) model. Future researchers should also develop a research model by adding new variables.

## Figures and Tables

**Table 1:** Measurement models.

Variables and Items	Outer Loadings	VIF
Entertainment [103] (Mean = 4.27, SD = 0.67, AVE = 0.707, C. alpha = 0.862, Rho_A = 0.869, CR = 0.906)		
ENT 1: The contents on social networks are believed to be thought-provoking in using sustainable products.	0.871	2.615
ENT 2: Using social networks is exciting.	0.881	2.452
ENT 3: Gathering data on sustainable services and sustainable products through social networks is fun.	0.819	2.047
ENT 4: Using social networks saves time in identifying sustainable products easily.	0.790	1.619
Customization [103] (Mean = 4.36, SD = 0.58, AVE = 0.597, C. alpha = 0.828, Rho_A = 0.839, CR = 0.879)		
CUS 1: Looking for tailored data related to sustainable products and services on social networks is possible.	0.701	1.507
CUS 2: Customized services are offered by social networks.	0.751	1.618
CUS 3: Social networks offer exciting feed data that users are interested in.	0.766	1.709
CUS 4: Using Social networks makes it easy to find sustainable products.	0.792	1.787
CUS 5: Social networks are everywhere.	0.848	2.231
Interaction [103] (Mean = 4.24, SD = 0.63, AVE = 0.666, C. alpha = 0.829, Rho_A = 0.859, CR = 0.887)		
INT 1: Conveying opinions between buyers/sellers through social networks is easy.	0.894	2.891
INT 2: Exchange of opinions or conversations related to sustainable products and services with buyers/sellers through social networks.	0.859	2.552
INT 3: Two-way interaction through social networks is easy.	0.849	1.899
INT 4: Sharing data with buyers/sellers through social networks is easy.	0.637	1.293
Word of mouth [103] (Mean = 4.34, SD = 0.67, AVE = 0.728, C. alpha = 0.813, Rho_A = 0.821, CR = 0.889)		
WOM 1: I like to share information about sustainable products or services from social networks with my friends.	0.890	2.056
WOM 2: I like uploading content from social networks on my page, blog, or microblog.	0.824	1.746
WOM 3: I like sharing thoughts on items or services acquired from social networks with my friends.	0.843	1.702
Trend [103] (Mean = 4.32, SD = 0.65, AVE = 0.771, C. alpha = 0.705, Rho_A = 0.724, CR = 0.871)		
TRE 1: It is a leading brand and supports sustainable products/services by using social networks.	0.903	1.422
TRE 2: Contents related to sustainable products/services on social networks are fresh.	0.852	1.422
eco-friendly consumers’ attitude [112] (Mean = 4.42, SD = 0.60, AVE = 0.700, C. alpha = 0.786, Rho_A = 0.802, CR = 0.875)		
ATT1: I would prefer to buy products that use biodegradable material in packaging.	0.782	1.513
ATT2: I would wish to buy those products that are picked up and recycled for other use.rend	0.852	1.716
ATT3: I would buy biodegradable products even if they belong to a less well-known company.	0.874	1.787
CSPB [88,105] (Mean = 4.35, SD = 0.63, AVE = 0.701, C. alpha = 0.787, Rho_A = 0.788, CR = 0.875)		
CSPB1: I would buy products with eco-friendly packaging in the near future.	0.853	1.708
CSPB2: I plan to buy eco-friendly packaged products regularly.	0.821	1.599
CSPB3: I intend to buy products with eco-friendly packaging due to my environmental concerns.	0.838	1.641

Notes: SD: Standard deviation; AVE: Average of Variance Extracted; C. alpha: Cronbach’s alpha; Rho_A, rho_A: reliability indices for each construct; CR: Composite Reliability; VIF: Variance Inflation Factor in items; level. CUS: Customization, INT: Interaction, WOM: Word of Mouth, ENT: Entertainment, TRE: Trend, ATT: Eco-friendly Attitude, CSPB: Customers’ sustainable Purchase Behavior

**Table 2:** Discriminant validity.

Constructs	CSPB	CUS	ENT	INT	TRE	WOM	ATT
CSPM	0.837						
CUS	0.723	0.773					
ENT	0.747	0.683	0.841				
INT	0.750	0.664	0.722	0.816			
TRE	0.730	0.756	0.726	0.755	0.878		
WOM	0.750	0.748	0.698	0.759	0.657	0.853	
ATT	0.744	0.646	0.692	0.658	0.624	0.658	0.837

Notes: Data in Table 2 are square roots of AVE (numbers in oblique line) and discriminant validity of a measurement model requires correlations between the constructs to be smaller than the square root of AVE; CSPB, CSPB; CUS, Customization; ENT, Entertainment; INT, Interaction; TRE, Trened; WOM, Word of mouth; ATT, eco-friendly consumers’ attitude.

**Table 3:** Results of research hypotheses and model fit.

Hypotheses	Direct Effect	Standard Deviation (SD)	T-Statistics	*p* Value	Low CI (Confidence Interval)	High CI (Confidence Interval)	Moderation	Decision
H1	0.354	0.036	9.921 ***	0.000	0.286	0.451		Supported
H2	0.121	0.045	2.707 **	0.007	0.463	0.612		Supported
H3	0.414	0.036	11.400 ***	0.000	0.782	0.869		Supported
H4	0.011	0.029	0.376	0.707	0.767	0.885		Not Supported
H5	0.014	0.032	0.431	0.667	0.644	0.802		Not Supported
H6a	0.070	0.048	1.472	0.142	0.315	0.478	No	Not Supported
H6b	0.043	0.043	1.000	0.318	0.141	0.325	No	Not Supported
H6c	0.188	0.070	2.667 **	0.008	0.285	0.410	Yes	Supported
H6d	0.138	0.040	3.462 ***	0.001			Yes	Supported
H6e	0.063	0.046	1.370	0.171			No	Not Supported
Model fit	R^2^	R^2^ Adjusted	Q^2^ predict					
CSPB	85.9%	85.6%	0.576					

Note: t > 1.96 at * *p* < 0.05; t > 2.58 at ** *p* < 0.01; t > 3.29 at *** *p* < 0.001; two-tailed test.

**Table 4:** PLS predict assessment of the manifest variable CPB.

Items	RMSE_PLS-SEM_	RMSE_LM_	∆RMSE
CSPB1	0.423	0.447	−0.024
CSPB2	0.529	0.534	−0.005
CSPB3	0.483	0.492	−0.009

Notes: RMSE = root mean squared error; gray-shaded results = PLS-SEM’s predictive power is lower than the LM benchmark.

**Table 5:** Bottleneck table and NCA effect sizes.

Bottleneck CSPB	ENT	CUS	INT	WOM	TRE	ATT
0	NN	NN	NN	NN	NN	NN
10	20.0	NN	7.1	9.1	NN	NN
20	26.7	35.3	28.6	18.2	28.6	20.0
30	26.7	35.3	28.6	18.2	42.9	50.0
40	26.7	35.3	42.9	45.5	57.1	50.0
50	26.7	35.3	42.9	45.5	57.1	50.0
60	26.7	35.3	42.9	45.5	57.1	50.0
70	26.7	64.7	42.9	45.5	57.1	50.0
80	60.0	64.7	57.1	54.5	71.4	60.0
90	60.0	64.7	57.1	72.7	71.4	60.0
100	86.7	76.5	78.6	72.7	71.4	70.0
NCA effect sizes (Accuracy and fit are 100%)						
Construct	CE-FDH	Slope				
ENT (Entertainment)	0.387 *	1.066				
CUS (Customization)	0.447 *	1.155				
INT (Interaction)	0.429 *	1.318				
WOM (Word of Mouth)	0.427 *	1.291				
TRE (Trend)	0.514 *	1.198				
ATT (Eco-friendly Attitude)	0.460 *	1.382				

Note: t > 1.96 at * *p* < 0.05; CE-FDH for CSPB, ceiling envelopment-free disposal hull.
